# On the Conservativeness of Robust Variance Estimators in Propensity Score Weighted Cox Models

**DOI:** 10.1002/pst.70130

**Published:** 2026-09-29

**Authors:** Hiroya Morita, Shunichiro Orihara, Fumitaka Shimizu, Masataka Taguri

**Affiliations:** ^1^ Department of Health Data Science Tokyo Medical University Tokyo Japan; ^2^ Department of Urology Juntendo University Graduate School of Medicine Tokyo Japan; ^3^ Department of Urology Juntendo Shizuoka Hospital Shizuoka Japan

**Keywords:** ATO, ATT, Cox regression models, inverse probability weighting, robust variance

## Abstract

In propensity score weighted analysis, robust variance that does not account for weight estimation (hereafter, uncorrected variance) is commonly used. In propensity score weighted Cox models (CoxPSW), the uncorrected variance is known to be conservative when weights for the average treatment effect (ATE) are used, but it remains unclear whether this conservativeness also holds for other weighting schemes. This study evaluated the performance of the uncorrected variance in CoxPSW when weights other than ATE are applied. We conducted an asymptotic comparison between the uncorrected variance and a variance estimator that accounts for weight estimation under non‐ATE weights. Their performance was further evaluated through simulation studies and real data analysis. The analytical results, simulations, and real data analysis indicated that the uncorrected variance is not necessarily conservative in CoxPSW when weights other than ATE are used. These findings suggest that variance estimators that account for weight estimation should be used when applying CoxPSW.

## 1 Introduction

Observational studies using real‐world data have become increasingly common. Because confounding is a major concern in observational research, it is essential to apply appropriate methods to address it. Propensity score weighted (PSW) analysis is one of the most widely used approaches [1, 2].

PSW typically employs weights derived from propensity scores. By applying different weights, PSW enables the estimation of various causal estimands, including the Average Treatment Effect (ATE), the Average Treatment Effect on the Treated (ATT), and the Average Treatment Effect for the Overlap population (ATO) [3]. For variance estimation, robust variance estimators that do not account for weight estimation (hereafter, uncorrected variance) are frequently used and serve as the default in many statistical software packages [1, 4, 5, 6]. For continuous outcomes, it is known that the uncorrected variance estimator is conservative for the ATE; however, recent studies report that this property does not always hold for other estimands, such as the ATT or ATO [7, 8].

In medical research, time‐to‐event outcomes are frequently analyzed. In the context of propensity score weighted Cox models (CoxPSW) [9], the uncorrected variance has been shown to be conservative when estimating the ATE [10]. However, the properties of the uncorrected variance estimator in CoxPSW for other estimands, such as the ATT and ATO, remain unclear. In this study, we investigate the properties of the uncorrected variance estimator in CoxPSW for estimands other than the ATE through mathematical derivation, simulation studies, and data analysis.

## 2 Extension of the Sandwich Variance to Weighting Schemes Other Than the ATE

We extend the asymptotic variance estimator accounting for weight estimation proposed by Shu et al. [10] to a general class of weights beyond the ATE.

Suppose we observe a random sample of n individuals from a study population. For each individual *i*, let $X_{i}$ denote a vector of baseline characteristics, and let $A_{i}\in \left\{0,1\right\}$ denote an indicator whether the individual received the treatment or not. Each individual is followed until either an event of interest or another event, denoted as censoring, occurs, and the observed time is defined as

$$T_{i}=\min\left(T_{i}^{*},C_{i}\right)$$

where $T_{i}^{*}$ represents the event time and $C_{i}$ the censoring time. We further define $\delta_{i}=I\left(T_{i}^{*}\leq C_{i}\right)$ as the event indicator. Throughout this work, we assume that, conditional on treatment assignment $A_{i}$, the censoring mechanism does not depend on the underlying event time $T_{i}^{*}$ nor the covariates $X_{i}$. We imposed this assumption because covariate‐dependent censoring could bias the point estimates. Addressing such censoring would require additional adjustment, such as inverse probability of censoring weighting (IPCW), as well as variance estimation accounting for the estimated censoring weights. Covariate‐dependent censoring and IPCW were not considered in this study. The observed data for each individual are $\left(X_{i},A_{i},T_{i},\delta_{i}\right)$. We assume that the standard causal inference assumptions hold: consistency, positivity, and conditional exchangeability.

Let $T_{a}^{*}$ denote the counterfactual time to failure that would have been observed for an individual had the treatment level been set to $A_{i}=a$. We consider fitting the proportional hazards model:

$$\lambda_{a}\left(t\right)=\lambda_{0}\left(t\right)\exp\left(\theta a\right),$$

where $\lambda_{a}\left(t\right)$ denotes the hazard function for the counterfactual failure time. The parameter θ presents the marginal log hazard ratio comparing the hazard under treatment with that under control. To obtain an estimate of θ, we adopt a PSW approach that reweights the observed sample. The estimator $\hat{\theta }$ is defined as the solution to the weighted score equation derived from the partial likelihood [11]:

$$\sum_{i=1}^{n}{\hat{w}}_{i}\delta_{i}\left[A_{i}-\frac{\sum_{\ell \in R_{i}}{\hat{w}}_{\ell }\exp\left(\theta A_{\ell }\right)A_{\ell }}{\sum_{\ell \in R_{i}}{\hat{w}}_{\ell }\exp\left(\theta A_{\ell }\right)}\right]=0.$$

Here, $R_{i}=\left\{\ell :T_{\ell }\geq T_{i},\delta_{i}=1\right\}$ denotes the risk set at the event time of individual i, and ${\hat{w}}_{i}$ represents the estimated weight for individual i. Although the model is misspecified, $\hat{\theta }$ converges to a well‐defined limiting value, $\theta^{*}$ as shown by Lin and Wei [12].

Let the weight be defined as a general function of the estimated propensity score, where the propensity score is defined as $e_{i}=P\left(A_{i}=1|X_{i}\right)$:

$$w_{i}=\frac{w\left(e_{i}\right)}{A_{i}e_{i}+\left(1-A_{i}\right)\left(1-e_{i}\right)}.$$

Here, $w\left(e_{i}\right)$ denotes an estimand‐specific weight function evaluated at the propensity score [3]. For instance, $w\left(e_{i}\right)=1$ for the ATE, $w\left(e_{i}\right)=e_{i}$ for the ATT, and $w\left(e_{i}\right)=e_{i}\left(1-e_{i}\right)$ for the ATO.

To account for the estimation of the propensity score, we consider a sandwich variance estimator that incorporates uncertainty from the design stage [13]. Following Shu et al., let γ denote the parameter vector in the propensity score model. Define $\beta ={\left(\theta ,\gamma^{⊤}\right)}^{⊤}$. The joint estimating equation for β can be written as

∑i=1nΦi(θ,γ)=∑i=1nψi(θ,γ)∑i=1nπi(γ)=∑i=1nwiδiAi−∑ℓ∈RiwℓexpθAℓAℓ∑ℓ∈RiwℓexpθAℓ∑i=1nAi−11+exp−γTXiXi=0

where the propensity score is estimated using the score function $\pi_{i}\left(\gamma \right)$ from the logistic model with 1 included in the vector of covariates $X_{i}$. The asymptotic variance estimator (hereafter, corrected variance) of $\hat{\beta }={\left(\hat{\theta },{\hat{\gamma }}^{⊤}\right)}^{⊤}$ is given by the following expression. The derivation follows the same steps as in Appendix A of Shu et al., which applies the arguments of Lin and Wei, and Binder [12, 14].

The corrected variance estimator is given by

$${\hat{\mathrm{var}}}_{\mathrm{CS}}\left(\hat{\beta }\right)=A{\left(\hat{\beta }\right)}^{-1}B\left(\hat{\beta }\right){\left\{A{\left(\hat{\beta }\right)}^{-1}\right\}}^{⊤},$$

where $A\left(\hat{\beta }\right)=-\sum_{i=1}^{n}\Phi_{i}^{\prime }\left(\hat{\beta }\right)$ and $B\left(\hat{\beta }\right)=\sum_{i=1}^{n}\Omega_{i}\left(\hat{\beta }\right)\Omega_{i}{\left(\hat{\beta }\right)}^{⊤}$. Here, the prime in the definition of $A\left(\hat{\beta }\right)$ denotes the first derivative. $\Omega_{i}\left(\hat{\beta }\right)$ is defined as

$$\Omega_{i}\left(\hat{\beta }\right)=\left(\begin{array}{l} \eta_{i}\left(\hat{\theta },\hat{\gamma }\right)\\ \pi_{i}\left(\hat{\gamma }\right) \end{array}\right),$$

where

$$\begin{array}{cc} \eta_{i}\left(\hat{\theta },\hat{\gamma }\right) & ={\hat{w}}_{i}\;\delta_{i}\left\{A_{i}-\frac{S_{1}\left(i\right)}{S_{0}\left(i\right)}\right\}-{\hat{w}}_{i}A_{i}\exp\left(A_{i}\hat{\theta }\right)\sum_{j=1}^{n}\delta_{j}{\hat{w}}_{j}\frac{I\left(T_{j}\leq T_{i}\right)}{S_{0}\left(j\right)}\\ & \;+{\hat{w}}_{i}\exp\left(A_{i}\hat{\theta }\right)\sum_{j=1}^{n}\delta_{j}{\hat{w}}_{j}I\left(T_{j}\leq T_{i}\right)\frac{S_{1}\left(j\right)}{S_{0}{\left(j\right)}^{2}}, \end{array}$$

$$S_{0}\left(i\right)=\sum_{l\in R_{i}}{\hat{w}}_{l}\exp\left(A_{l}\hat{\theta }\right),$$

$$S_{1}\left(i\right)=\sum_{l\in R_{i}}{\hat{w}}_{l}\exp\left(A_{l}\hat{\theta }\right)A_{l}$$

The corrected variance is essentially identical to that presented by Zhao et al. for the case of a continuous treatment variable [15].

## 3 Conservativeness Under Non‐ATE Weighting

As noted above, when using ATE weights $w\left(e_{i}\right)\equiv 1$, the uncorrected variance is conservative. In the Supporting Information, we show that for general weight functions other than those corresponding to the ATE, the uncorrected variance is not necessarily asymptotically conservative. This finding is consistent with previous results for continuous outcomes [8].

## 4 Simulation Study

Following the simulation framework for continuous outcomes [8], we generated 10,000 independent datasets, each consisting of *n* = 300, 1000, or 10,000 independent and identically distributed observations. For each dataset, we simulated three covariates (*L*_1_, *L*_2_, *L*_3_), a treatment indicator (*A*), event status (δ) and a recorded time (*T*).

$$L_{1i}∼\mathrm{Ber}\left(0.5\right),L_{2i}∼N\left(0,1\right),L_{3i}∼N\left(0,1\right),$$

$$A_{i}∼\mathrm{Ber}\left(\frac{1}{1+\exp\left(2+0.5L_{1i}-\alpha_{1}L_{2i}-\alpha_{2}L_{3i}\right)}\right),$$

$$T_{0i}=\text{Exp}\left(0.01\right),$$

$$\eta_{i}=\log\left(0.8\right)A_{i}+\log\left(0.4\right)L_{1i}+\log\left(5\right)A_{i}L_{1i}+\log\left(\beta_{1}\right)L_{2i}+\log\left(\beta_{2}\right)L_{3i},$$

$$T_{i}^{*}=T_{0i}\exp\left(-\eta_{i}\right),$$

$$C_{i}=\text{Exp}\left(0.001\right),$$

$$\delta_{i}=I\left(T_{i}^{*}\leq C_{i}\right),$$

$$T_{i}=\min\left(T_{i}^{*},C_{i}\right)$$

$\alpha_{1},\alpha_{2},\beta_{1},\beta_{2}$ denote parameters that differ across simulation settings. The two scenarios are presented in Table 1. In the first scenario, confounders were more strongly associated with the outcome than treatment, whereas in the second scenario, confounders were more strongly associated with the treatment than outcome. The censoring rate was approximately 20%.

**TABLE 1:** Simulation scenarios.

Scenario	$\alpha_{1}$	$\alpha_{2}$	$\beta_{1}$	$\beta_{2}$
Scenario 1	−0.1	−0.1	0.4	0.4
Scenario 2	0.5	0.5	0.95	0.95

For each scenario presented in Table 1, CoxPSW were applied to estimate ATE, ATT, and ATO. For variance estimation, we compared the uncorrected variance estimator with the corrected variance estimator in terms of coverage probability and the mean of standard error (SE). The true hazard ratio was approximated by the estimated hazard ratio obtained from a simulation with *n* = 50,000,000 for each estimand.

The results are presented in Tables 2 and 3. In the first scenario, the uncorrected variance estimator was conservative regardless of the weighting scheme used. In contrast, in the second scenario, the coverage probability of the uncorrected variance estimator fell well below 95% when ATT (0.915) and ATO (0.923) weights were used even when *n* = 10,000. The corrected variance achieved approximately 95% coverage. Similar patterns were observed for the mean SEs. In the first scenario, the uncorrected variance estimator yielded larger SEs on average across all weighting schemes. In the second scenario, the mean SEs based on the uncorrected variance estimator were smaller for the ATT and ATO, whereas they were slightly larger for the ATE. Additional simulation results obtained under alternative parameter settings are presented in the Supporting Information. Similar trends were observed across these settings.

**TABLE 2:** Simulation results for Scenario 1.

Weight		True log HR	Bias of log HR		Mean of SE		Coverage probability	
	*N*			Emp SE	Uncorrected variance	Corrected variance	Uncorrected variance	Corrected variance
ATE	300	0.3308	0.008	0.201	0.225	0.173	0.964	0.907
ATE	1000	0.3308	0.004	0.101	0.124	0.096	0.980	0.936
ATE	10,000	0.3308	0.000	0.031	0.039	0.031	0.986	0.946
ATT	300	0.2285	0.000	0.184	0.208	0.173	0.967	0.932
ATT	1000	0.2285	0.000	0.097	0.115	0.095	0.978	0.945
ATT	10,000	0.2285	0.001	0.031	0.036	0.030	0.982	0.945
ATO	300	0.2393	−0.000	0.181	0.209	0.169	0.972	0.932
ATO	1000	0.2393	0.001	0.097	0.115	0.094	0.979	0.940
ATO	10,000	0.2393	0.001	0.030	0.037	0.030	0.981	0.946

Abbreviations: Emp SE, empirical standard error; HR, hazard ratio; log, natural logarithm; *N*, sample size, SE, standard error.

**TABLE 3:** Simulation results for Scenario 2.

Weight		True log HR	Bias of log HR		Mean of SE		Coverage probability	
	*N*			Emp SE	Uncorrected variance	Corrected variance	Uncorrected variance	Corrected variance
ATE	300	0.5440	−0.009	0.223	0.201	0.189	0.926	0.906
ATE	1000	0.5440	−0.003	0.115	0.113	0.110	0.945	0.935
ATE	10,000	0.5440	−0.002	0.036	0.036	0.036	0.950	0.945
ATT	300	0.3951	−0.002	0.233	0.197	0.223	0.902	0.939
ATT	1000	0.3951	−0.003	0.122	0.107	0.122	0.914	0.950
ATT	10,000	0.3951	0.000	0.039	0.034	0.038	0.915	0.948
ATO	300	0.4190	−0.000	0.220	0.192	0.210	0.908	0.939
ATO	1000	0.4190	−0.003	0.116	0.105	0.115	0.923	0.948
ATO	10,000	0.4190	0.000	0.037	0.033	0.036	0.923	0.949

Abbreviations: Emp SE, empirical standard error; HR, hazard ratio; log, natural logarithm; *N*, sample size; SE, standard error.

## 5 Real Data Analysis

In this section, we provide an illustrative example using real‐world data on muscle‐invasive bladder cancer [16]. Our analysis focused on estimating the causal effects of adjuvant therapy on cancer‐specific mortality. The study sample consisted of 322 patients, including 74 in the adjuvant chemotherapy group (treatment group) and 248 in the radical cystectomy alone group (control group). Cancer‐specific death occurred in 23 patients (31.1%) in the treatment group and in 45 patients (18.2%) in the control group. The covariates used in the analysis are described in the Supporting Information. In this study, we conducted analyses for the overall population as well as subgroup analyses [16]. For each subgroup, analyses were performed using ATE, ATT, and ATO weighting. Variances were estimated using the uncorrected variance estimator and the corrected variance estimator.

The results are presented in Table 4. For the overall population analysis, the uncorrected variance estimator yielded more conservative results across all three weighting schemes. In contrast, in the subgroup analyses, there were several subgroups in which the uncorrected variance estimator produced smaller variance estimates for ATT or ATO weighting compared with the corrected variance.

**TABLE 4:** Results of the data analysis.

Weight	Subgroup	log HR	Uncorrected SE	Corrected SE	Uncorrected SE/Corrected SE
ATE	Overall population	−0.148	0.287	0.237	1.211
ATE	Age (years) 70 ≥	−0.661	0.440	0.366	1.202
ATE	Age (years) 70 <	−0.161	0.419	0.380	1.103
ATE	Male	−0.486	0.329	0.260	1.265
ATE	Female	0.162	0.736	0.703	1.047
ATE	NAC present	0.260	0.618	0.538	1.149
ATE	NAC absent	−0.505	0.330	0.261	1.264
ATE	Pathological T Stage 3 ≥	−0.421	0.287	0.259	1.108
ATE	Pathological T Stage 2 ≤	−1.038	0.687	0.618	1.112
ATE	Lymph nodes metastasis present	−1.569	0.404	0.393	1.028
ATE	Lymph nodes metastasis absent	−0.121	0.379	0.302	1.255
ATE	Venous invasion present	−0.701	0.336	0.288	1.167
ATE	Venous invasion absent	−0.399	0.541	0.495	1.093
ATE	Lymphatic duct invasion present	−0.760	0.318	0.272	1.169
ATE	Lymphatic duct invasion absent	−0.010	0.553	0.503	1.099
ATT	Overall population	−0.485	0.257	0.237	1.084
ATT	Age (years) 70 ≥	−0.731	0.334	0.321	1.040
ATT	Age (years) 70 <	−0.416	0.360	0.339	1.062
ATT	Male	−0.543	0.272	0.250	1.088
ATT	Female	−0.101	0.606	0.611	0.992
ATT	NAC present	−0.228	0.617	0.588	1.049
ATT	NAC absent	−0.543	0.275	0.250	1.100
ATT	Pathological T Stage 3 ≥	−0.647	0.279	0.267	1.045
ATT	Pathological T Stage 2 ≤	−0.361	0.670	0.668	1.003
ATT	Lymph nodes metastasis present	−1.733	0.426	0.421	1.012
ATT	Lymph nodes metastasis absent	0.149	0.343	0.326	1.052
ATT	Venous invasion present	−0.765	0.283	0.278	1.018
ATT	Venous invasion absent	0.126	0.492	0.510	0.965
ATT	Lymphatic duct invasion present	−0.872	0.287	0.281	1.021
ATT	Lymphatic duct invasion absent	0.753	0.511	0.531	0.962
ATO	Overall population	−0.322	0.260	0.233	1.116
ATO	Age (years) 70 ≥	−0.619	0.339	0.309	1.097
ATO	Age (years) 70 <	−0.145	0.377	0.355	1.062
ATO	Male	−0.38	0.277	0.244	1.135
ATO	Female	0.246	0.613	0.617	0.994
ATO	NAC present	0.094	0.598	0.527	1.135
ATO	NAC absent	−0.372	0.281	0.249	1.129
ATO	Pathological T Stage 3 ≥	−0.428	0.282	0.256	1.102
ATO	Pathological T Stage 2 ≤	−0.335	0.650	0.631	1.030
ATO	Lymph nodes metastasis present	−1.621	0.421	0.410	1.027
ATO	Lymph nodes metastasis absent	0.185	0.335	0.308	1.088
ATO	Venous invasion present	−0.605	0.299	0.271	1.103
ATO	Venous invasion absent	0.127	0.482	0.484	0.996
ATO	Lymphatic duct invasion present	−0.705	0.296	0.265	1.117
ATO	Lymphatic duct invasion absent	0.660	0.503	0.501	1.004

Abbreviations: HR, hazard ratio; log, natural logarithm; *N*, sample size; NAC, neoadjuvant chemotherapy; SE, standard error.

## 6 Discussion

In this study, we showed that the uncorrected variance estimator that ignores the estimation of weights in CoxPSW is not necessarily conservative for general weighting schemes other than the ATE. This result is consistent with previous findings for continuous outcomes [7, 8].

The simulation results demonstrated that the uncorrected variance estimator may fail to be conservative when ATT or ATO weights are used. A general pattern observed in the simulations was that when covariates were weakly associated with the exposure but strongly associated with the outcome, the uncorrected variance estimator tended to be conservative regardless of the weighting scheme. Similar phenomena related to prognostic variables have been reported for continuous outcomes [17]. Further research is needed to clarify the theoretical properties of the asymptotic variance of CoxPSW.

In the real data analysis, we also compared the uncorrected variance estimator with the corrected variance estimator. Because the theory of the uncorrected variance estimator is based on asymptotic variance, its properties are not necessarily guaranteed in finite samples. In the present data analysis, when ATE weights were used, the uncorrected variance estimator produced larger estimates across all subgroups, as expected. In contrast, when ATT or ATO weights were used, the uncorrected variance estimator produced smaller estimates in some subgroups.

In conclusion, similar to the cases of continuous outcomes, the uncorrected variance estimator in CoxPSW is not guaranteed to be conservative when weighting schemes other than the ATE are used. In general, regardless of whether the uncorrected variance estimator is conservative, variance estimators that account for the estimation of the weights should be used when applying CoxPSW.

## Funding

This work was partially supported by JSPS KAKENHI Grant Number JP24K14862.

## Ethics Statement

The authors have nothing to report.

## Consent

The authors have nothing to report.

## Conflicts of Interest

The authors declare no conflicts of interest.

## Supporting information


**Table S1:** Simulation scenarios.
**Table S2:** Simulation results for Scenario 3.
**Table S3:** Simulation results for Scenario 4.
**Table S4:** Simulation results for Scenario 5.
**Table S5:** Simulation results for Scenario 6.
**Table S6:** Simulation results for Scenario 7.
**Table S7:** Simulation results for Scenario 8.
**Table S8:** Simulation results for Scenario 9.
**Table S9:** Simulation results for Scenario 10.
**Table S10:** Simulation results for Scenario 11.

## Data Availability

Research data are not shared. The R code is available at the following URL: https://github.com/hirmorita/On‐the‐Conservativeness‐of‐Robust‐Variance‐Estimators‐in‐Propensity‐Score‐Weighted‐Cox‐Models_Rcode.
