# Supplementary material for: On the Conservativeness of Robust Variance Estimators in Propensity Score Weighted Cox Models
Source: Pharm Stat. 2026 Sep 29;25(6):e70130. doi: 10.1002/pst.70130 (PMC13621226; doi:10.1002/pst.70130)
Supplement: Supplementary file 1 [file PST-25-0-s001.docx]

**Supplemental File**

**Comparison of Asymptotic Variances under General Weights**

In Shu et al.^1^, it is shown that for CoxPSW using ATE weights, the robust variance (hereafter, uncorrected variance) is asymptotically larger than the corrected sandwich variance, meaning that the uncorrected variance is conservative. Here, we show that for other types of weights, the uncorrected variance is not necessarily conservative in general. The notation used in this study follows Appendix A–C of Shu et al. Furthermore, the relationship between the uncorrected variance and the corrected variance used in the proof is based on the result in Appendix B of Shu et al.

We denote the uncorrected variance estimator by $\hat{var}_{R}\left( \hat{\theta} \right)$ and the corrected variance estimator by $\hat{var}_{CS}\left( \hat{\boldsymbol{\beta}} \right)$. The estimator $\hat{var}_{CS}\left( \hat{\boldsymbol{\beta}} \right)$ is defined as described in the main text. The uncorrected variance estimator $\hat{var}_{R}\left( \hat{\theta} \right)$ is given by the following expression.

$$\hat{var}_{R}\left( \hat{\theta} \right)=A\left( \hat{\theta} \right)^{-1}B\left( \hat{\theta} \right)\left\{ A\left( \hat{\theta} \right)^{-1} \right\}^{\top}$$

where

$A\left( \hat{\theta} \right)=-\sum_{i=1}^{n} \psi_{i}^{'}\left( \hat{\theta} \right),$

$B\left( \hat{\theta} \right)=\sum_{i=1}^{n} \eta_{i}\left( \hat{\theta} \right){\eta_{i}\left( \hat{\theta} \right)}^{\top},$

$$\eta_{i}\left( \hat{\theta} \right)=\hat{w}_{i}\text{ }\delta_{i}\left\{ A_{i} - \frac{S_{1}\left( i \right)}{S_{0}\left( i \right)} \right\}-\hat{w}_{i}A_{i}\exp\left( A_{i}\hat{\theta} \right)\sum_{j=1}^{n} \delta_{j}\hat{w}_{j}\frac{I\left( T_{j}\leq T_{i} \right)}{S_{0}\left( j \right)}+\hat{w}_{i}\exp\left( A_{i}\hat{\theta} \right)\sum_{j=1}^{n} \delta_{j}\hat{w}_{j}I\left( T_{j}\leq T_{i} \right)\frac{S_{1}\left( j \right)}{S_{0}(j)^{2}},$$

$S_{0}(i)=\sum_{l\in\mathcal{R}_{i}} \hat{w}_{l}\exp(A_{l}\hat{\theta})$,

$S_{1}(i)=\sum_{l\in\mathcal{R}_{i}} \hat{w}_{l}\exp(A_{l}\hat{\theta})A_{l}$.

Here, the prime in the definition of $A\left( \hat{\theta} \right)$denotes the first derivative.

The relationship between the correct variance and the uncorrected variance can be expressed as:

$${n\hat{var}}_{CS}\left( \hat{\theta} \right)={n\hat{var}}_{R}\left( \hat{\theta} \right)+\frac{n^{2}}{\left( A_{11} \right)^{2}}\left[ {\hat{\boldsymbol{d}}}^{\top}\frac{1}{n}\sum_{i=1}^{n} \left\{ \left( A_{i}-\hat{e}_{i}^{2} \right)\boldsymbol{X}_{i}\boldsymbol{X}_{i}^{\top} \right\}\hat{\boldsymbol{d}}+2{\hat{\boldsymbol{d}}}^{\top}\frac{1}{n}\sum_{i=1}^{n} \hat{w}_{i}L_{0i}\left( A_{i}-\hat{e}_{i} \right)\boldsymbol{X}_{i} \right]$$

where $A\left( \hat{\boldsymbol{\beta}} \right)=-\sum_{i=1}^{n} {\Phi^{'}}_{i}\left( \hat{\boldsymbol{\beta}} \right)=-\sum_{i=1}^{n} \left[ \begin{matrix} \frac{\partial\psi_{i}(\hat{\boldsymbol{\beta}})}{\partial\hat{\theta}} & \frac{\partial\psi_{i}(\hat{\boldsymbol{\beta}})}{\partial{\hat{\boldsymbol{\gamma}}}^{\top}} \\ \frac{\partial\pi_{i}(\hat{\boldsymbol{\gamma}})}{\partial\hat{\theta}} & \frac{\partial\pi_{i}(\hat{\boldsymbol{\gamma}})}{\partial{\hat{\boldsymbol{\gamma}}}^{\top}} \end{matrix} \right]=\left[ \begin{matrix} A_{11} & A_{12} \\ 0 & A_{22} \end{matrix} \right]$,

where $\hat{var}_{CS}\left( \hat{\theta} \right)$ is given by the element in the first row and first column of the matrix $\hat{var}_{CS}\left( \hat{\boldsymbol{\beta}} \right)$,

$\hat{\boldsymbol{d}}={\hat{\boldsymbol{U}}}^{-1}\{\frac{1}{n}\sum_{i=1}^{n} k_{i}\hat{L}_{0i}\boldsymbol{X}_{i}\}$, $k_{i}=A_{i}\left( 1-\hat{e}_{i} \right)\left( {w(\hat{e}_{i})}^{'}-\frac{w(\hat{e}_{i})}{\hat{e}_{i}} \right)+\left( 1-A_{i} \right)\hat{e}_{i}\left( {w(\hat{e}_{i})}^{'}+\frac{w(\hat{e}_{i})}{{1-\hat{e}}_{i}} \right)$,

$\hat{\boldsymbol{U}}=\frac{1}{n}\sum_{i=1}^{n} \hat{e}_{i}\left( 1-\hat{e}_{i} \right)\boldsymbol{X}_{i}\boldsymbol{X}_{i}^{\top}$, and $L_{0i}=\delta_{i}\left\{ A_{i}-\frac{S_{1}\left( i \right)}{S_{2}\left( i \right)} \right\}-exp(\hat{\theta}A_{i})[\sum_{j=1}^{n} \frac{\hat{w}_{j}\delta_{j}I(T_{j}\leq T_{i})}{S_{0}(j)}\{A_{i}-\frac{S_{1}\left( i \right)}{S_{2}\left( i \right)}\}$

Here, which asymptotic variance is larger is determined by the sign of the final term. Rearranging the part that determines the sign of the final term in the same way as in Shu et al., we obtain the following expression:

$${\hat{\boldsymbol{d}}}^{\top}\frac{1}{n}\sum_{i=1}^{n} \left\{ \left( A_{i}-\hat{e}_{i}^{2} \right)\boldsymbol{X}_{i}\boldsymbol{X}_{i}^{T} \right\}\hat{d}+2{\hat{\boldsymbol{d}}}^{\top}\frac{1}{n}\sum_{i=1}^{n} \hat{w}_{i}L_{0i}\left( A_{i}-\hat{e}_{i} \right)\boldsymbol{X}_{i}$$

$${=\left\{ \frac{1}{n}\sum_{i=1}^{n} k_{i}\hat{L}_{0i}\boldsymbol{X}_{i} \right\}}^{\top}{\hat{\boldsymbol{U}}}^{-1}\frac{1}{n}\sum_{i=1}^{n} \left\{ \left( A_{i}-\hat{e}_{i}^{2} \right)\boldsymbol{X}_{i}\boldsymbol{X}_{i}^{\top} \right\}{\hat{\boldsymbol{U}}}^{-1}\left\{ \frac{1}{n}\sum_{i=1}^{n} k_{i}\hat{L}_{0i}\boldsymbol{X}_{i} \right\}+2\left\{ \frac{1}{n}\sum_{i=1}^{n} k_{i}\hat{L}_{0i}\boldsymbol{X}_{i} \right\}^{\top}{\hat{\boldsymbol{U}}}^{-1}\frac{1}{n}\sum_{i=1}^{n} \hat{w}_{i}\hat{L}_{0i}\left( A_{i}-\hat{e}_{i} \right)\boldsymbol{X}_{i}$$

$$\begin{aligned} \approx\left\{ \frac{1}{n}\sum_{i=1}^{n} k_{i}\hat{L}_{0i}\boldsymbol{X}_{i} \right\}^{\top}\hat{U}^{-1}\left\{ \frac{1}{n}\sum_{i=1}^{n} k_{i}\hat{L}_{0i}\boldsymbol{X}_{i} \right\}+2\left\{ \frac{1}{n}\sum_{i=1}^{n} k_{i}\hat{L}_{0i}\boldsymbol{X}_{i} \right\}^{\top}\hat{U}^{-1}\frac{1}{n}\sum_{i=1}^{n} \hat{w}_{i}\hat{L}_{0i}\left( A_{i}-\hat{e}_{i} \right)X_{i}\#\left( 1 \right) \end{aligned}$$

where $A_{n}\approx B_{n}$ means that $A_{n}-B_{n}=o_{p}(1)$ for sequences of random variables $A_{n}$ and $B_{n}$. We used $U_{n}\approx\frac{1}{n}\sum_{i=1}^{n} \left\{ \left( A_{i}-\hat{e}_{i}^{2} \right)\boldsymbol{X}_{i}\boldsymbol{X}_{i}^{\top} \right\}$.

In the ATE case, the relationship $\hat{w}_{i}\left( A_{i}-\hat{e}_{i} \right)=-k_{i}$ holds. In the case of the ATE, this expression simplifies to the following form. Equation (1) can be rearranged as follows.

$${-\left\{ \frac{1}{n}\sum_{i=1}^{n} k_{i}\hat{L}_{0i}\boldsymbol{X}_{i} \right\}}^{\top}\hat{U}^{-1}\left\{ \frac{1}{n}\sum_{i=1}^{n} k_{i}\hat{L}_{0i}\boldsymbol{X}_{i} \right\}\leq0$$

Therefore, the expression becomes a quadratic form, which is always non-positive. However, for weights other than ATE, the sign of the above expression is not determined in general.

For example, the expressions for the ATT and ATO are given as follows. In the case of the ATT, $w\left( \hat{e}_{i} \right)=\hat{e}_{i}$. Then, $k_{i}=A_{i}\left( 1-\hat{e}_{i} \right)\left( {w(\hat{e}_{i})}^{'}-\frac{w(\hat{e}_{i})}{\hat{e}_{i}} \right)+\left( 1-A_{i} \right)\hat{e}_{i}\left( {w(\hat{e}_{i})}^{'}+\frac{w(\hat{e}_{i})}{{1-\hat{e}}_{i}} \right)=\left( 1-A_{i} \right)\frac{\hat{e}_{i}}{(1-\hat{e}_{i})}$, and $\hat{w}_{i}\left( A_{i}-\hat{e}_{i} \right)=A\left( 1-\hat{e}_{i} \right)-(1-A)(\frac{{\hat{e}^{2}}_{i}}{1-\hat{e}_{i}})$. Thus, the sign is not uniquely determined.

In the case of the ATO, $w\left( \hat{e}_{i} \right)=\hat{e}_{i}(1-\hat{e}_{i})$. Then, $k_{i}=-A_{i}\hat{e}_{i}(1-\hat{e}_{i})+\left( 1-A_{i} \right)\hat{e}_{i}(1-\hat{e}_{i})$, and $\hat{w}_{i}\left( A_{i}-\hat{e}_{i} \right)=A\left( 1-\hat{e}_{i} \right)-(1-A)(\frac{{\hat{e}^{2}}_{i}}{1-\hat{e}_{i}})$. Thus, in this case as well, the sign is not uniquely determined.

Consequently, the relative magnitude of the asymptotic variances depends on the specific setting.

**References**

1.	Shu D, Young JG, Toh S, Wang R. Variance estimation in inverse probability weighted Cox models. *Biometrics*. 2021;77(3):1101-1117.

**Covariates used in the Real-World Data analysis**

In the present analysis, the propensity score was estimated using the following eight covariates: age (continuous), sex, local tumor stage, lymph node status (positive or negative), type of urinary diversion, neoadjuvant chemotherapy (yes or no), venous invasion (yes or no), and lymphatic duct invasion (yes or no). Subgroup analyses stratified by each of these covariates were also conducted.

**Additional simulation scenarios**

We conducted additional simulation analyses under Scenarios 3–11 using the parameter settings presented in Table S1. Scenarios 3–5 represented settings with stronger associations of the covariate with both treatment assignment and the outcome than in Scenarios 1 and 2, corresponding to stronger confounding. Scenarios 6 and 7 represented settings in which the association with the outcome was kept the same as in Scenarios 1 and 2, while the probability of treatment assignment was higher. Scenarios 8–11 had outcome-model settings similar to those in Scenarios 3–5 but a lower probability of treatment assignment. The results are presented in Tables S2–S10. Except for Scenario 7, the uncorrected variance estimator yielded conservative results for ATE, ATT, and ATO.

**Table S1** Simulation scenarios

| Scenario | $\alpha_{1}$ | $\alpha_{2}$ | $\beta_{1}$ | $\beta_{2}$ |
| --- | --- | --- | --- | --- |
| Scenario 3 | -1 | -1 | 5 | 5 |
| Scenario 4 | 1 | 1 | 5 | 5 |
| Scenario 5 | -1 | -1 | 3 | 3 |
| Scenario 6 | 1 | 1 | 0.4 | 0.4 |
| Scenario 7 | 1 | 1 | 0.95 | 0.95 |
| Scenario 8 | -0.1 | -0.1 | 5 | 5 |
| Scenario 9 | 0.5 | 0.5 | 5 | 5 |
| Scenario 10 | -0.1 | -0.1 | 3 | 3 |
| Scenario 11 | 0.5 | 0.5 | 3 | 3 |

**Table S2** Simulation results for scenario 3

|  |  |  |  |  | Mean of SE | |  | Coverage probability | |
| --- | --- | --- | --- | --- | --- | --- | --- | --- | --- |
| Weight | N | True log HR | Bias of  log HR | Emp SE | Uncorrected  variance | Corrected variance |  | Uncorrected  variance | Corrected variance |
| ATE | 300 | 0.2304 | -0.079 | 0.198 | 0.227 | 0.155 |  | 0.961 | 0.863 |
| ATE | 1000 | 0.2304 | -0.016 | 0.127 | 0.144 | 0.102 |  | 0.964 | 0.878 |
| ATE | 10000 | 0.2304 | 0.000 | 0.042 | 0.052 | 0.040 |  | 0.985 | 0.941 |
| ATT | 300 | 0.2183 | -0.012 | 0.236 | 0.278 | 0.216 |  | 0.982 | 0.941 |
| ATT | 1000 | 0.2183 | -0.012 | 0.127 | 0.154 | 0.119 |  | 0.983 | 0.938 |
| ATT | 10000 | 0.2183 | -0.001 | 0.039 | 0.049 | 0.038 |  | 0.981 | 0.940 |
| ATO | 300 | 0.2446 | -0.013 | 0.168 | 0.226 | 0.167 |  | 0.992 | 0.950 |
| ATO | 1000 | 0.2446 | 0.000 | 0.086 | 0.122 | 0.091 |  | 0.991 | 0.961 |
| ATO | 10000 | 0.2446 | -0.003 | 0.028 | 0.039 | 0.029 |  | 0.989 | 0.949 |

Abbreviations: Emp SE, empirical standard error; HR, hazard ratio; log, natural logarithm; N, sample size, SE, standard error

**Table S3** Simulation results for scenario 4

|  |  |  |  |  | Mean of SE | |  | Coverage probability | |
| --- | --- | --- | --- | --- | --- | --- | --- | --- | --- |
| Weight | N | True log HR | Bias of  log HR | Emp SE | Uncorrected  variance | Corrected variance |  | Uncorrected  variance | Corrected variance |
| ATE | 300 | 0.2291 | 0.119 | 0.400 | 0.313 | 0.257 |  | 0.796 | 0.734 |
| ATE | 1000 | 0.2291 | 0.043 | 0.244 | 0.216 | 0.188 |  | 0.872 | 0.836 |
| ATE | 10000 | 0.2291 | 0.011 | 0.091 | 0.088 | 0.080 |  | 0.929 | 0.912 |
| ATT | 300 | 0.1740 | 0.020 | 0.124 | 0.190 | 0.118 |  | 0.997 | 0.937 |
| ATT | 1000 | 0.1740 | 0.003 | 0.067 | 0.106 | 0.064 |  | 0.996 | 0.951 |
| ATT | 10000 | 0.1740 | 0.002 | 0.020 | 0.034 | 0.020 |  | 1.000 | 0.960 |
| ATO | 300 | 0.1996 | 0.007 | 0.135 | 0.190 | 0.128 |  | 0.990 | 0.932 |
| ATO | 1000 | 0.1996 | 0.002 | 0.074 | 0.105 | 0.071 |  | 0.996 | 0.938 |
| ATO | 10000 | 0.1996 | -0.001 | 0.022 | 0.033 | 0.023 |  | 0.992 | 0.952 |

Abbreviations: Emp SE, empirical standard error; HR, hazard ratio; log, natural logarithm; N, sample size, SE, standard error

**Table S4** Simulation results for scenario 5

|  |  |  |  |  | Mean of SE | |  | Coverage probability | |
| --- | --- | --- | --- | --- | --- | --- | --- | --- | --- |
| Weight | N | True log HR | Bias of  log HR | Emp SE | Uncorrected  variance | Corrected variance |  | Uncorrected  variance | Corrected variance |
| ATE | 300 | 0.2976 | -0.074 | 0.199 | 0.207 | 0.150 |  | 0.930 | 0.859 |
| ATE | 1000 | 0.2976 | -0.021 | 0.110 | 0.129 | 0.095 |  | 0.969 | 0.901 |
| ATE | 10000 | 0.2976 | -0.002 | 0.038 | 0.047 | 0.037 |  | 0.984 | 0.945 |
| ATT | 300 | 0.2686 | -0.015 | 0.243 | 0.258 | 0.223 |  | 0.962 | 0.940 |
| ATT | 1000 | 0.2686 | -0.003 | 0.130 | 0.145 | 0.125 |  | 0.976 | 0.945 |
| ATT | 10000 | 0.2686 | -0.003 | 0.040 | 0.047 | 0.041 |  | 0.976 | 0.951 |
| ATO | 300 | 0.3035 | 0.001 | 0.175 | 0.207 | 0.169 |  | 0.981 | 0.942 |
| ATO | 1000 | 0.3035 | -0.002 | 0.090 | 0.112 | 0.092 |  | 0.984 | 0.959 |
| ATO | 10000 | 0.3035 | -0.001 | 0.030 | 0.035 | 0.029 |  | 0.977 | 0.944 |

Abbreviations: Emp SE, empirical standard error; HR, hazard ratio; log, natural logarithm; N, sample size, SE, standard error

**Table S5** Simulation results for scenario 6

|  |  |  |  |  | Mean of SE | |  | Coverage probability | |
| --- | --- | --- | --- | --- | --- | --- | --- | --- | --- |
| Weight | N | True log HR | Bias of  log HR | Emp SE | Uncorrected  variance | Corrected variance |  | Uncorrected  variance | Corrected variance |
| ATE | 300 | 0.3319 | -0.065 | 0.197 | 0.204 | 0.154 |  | 0.939 | 0.865 |
| ATE | 1000 | 0.3319 | -0.023 | 0.108 | 0.124 | 0.095 |  | 0.965 | 0.913 |
| ATE | 10000 | 0.3319 | -0.003 | 0.040 | 0.046 | 0.037 |  | 0.977 | 0.931 |
| ATT | 300 | 0.2927 | -0.016 | 0.239 | 0.252 | 0.226 |  | 0.967 | 0.942 |
| ATT | 1000 | 0.2927 | -0.004 | 0.134 | 0.141 | 0.127 |  | 0.958 | 0.940 |
| ATT | 10000 | 0.2927 | -0.001 | 0.042 | 0.046 | 0.041 |  | 0.969 | 0.943 |
| ATO | 300 | 0.3319 | -0.009 | 0.173 | 0.200 | 0.171 |  | 0.977 | 0.944 |
| ATO | 1000 | 0.3319 | 0.000 | 0.092 | 0.109 | 0.093 |  | 0.979 | 0.955 |
| ATO | 10000 | 0.3319 | -0.002 | 0.028 | 0.034 | 0.029 |  | 0.985 | 0.957 |

Abbreviations: Emp SE, empirical standard error; HR, hazard ratio; log, natural logarithm; N, sample size, SE, standard error

**Table S6** Simulation results for scenario 7

|  |  |  |  |  | Mean of SE | |  | Coverage probability | |
| --- | --- | --- | --- | --- | --- | --- | --- | --- | --- |
| Weight | N | True log HR | Bias of  log HR | Emp SE | Uncorrected  variance | Corrected variance |  | Uncorrected  variance | Corrected variance |
| ATE | 300 | 0.5430 | -0.016 | 0.248 | 0.206 | 0.195 |  | 0.914 | 0.887 |
| ATE | 1000 | 0.5430 | -0.004 | 0.147 | 0.129 | 0.126 |  | 0.921 | 0.917 |
| ATE | 10000 | 0.5430 | 0.002 | 0.048 | 0.046 | 0.046 |  | 0.948 | 0.946 |
| ATT | 300 | 0.4244 | 0.006 | 0.234 | 0.207 | 0.222 |  | 0.911 | 0.931 |
| ATT | 1000 | 0.4244 | 0.002 | 0.128 | 0.116 | 0.124 |  | 0.919 | 0.950 |
| ATT | 10000 | 0.4244 | 0.001 | 0.039 | 0.037 | 0.039 |  | 0.940 | 0.954 |
| ATO | 300 | 0.4603 | 0.002 | 0.201 | 0.186 | 0.194 |  | 0.933 | 0.940 |
| ATO | 1000 | 0.4603 | 0.001 | 0.110 | 0.102 | 0.107 |  | 0.935 | 0.950 |
| ATO | 10000 | 0.4603 | -0.001 | 0.033 | 0.032 | 0.034 |  | 0.939 | 0.950 |

Abbreviations: Emp SE, empirical standard error; HR, hazard ratio; log, natural logarithm; N, sample size, SE, standard error

**Table S7** Simulation results for scenario 8

|  |  |  |  |  | Mean of SE | |  | Coverage probability | |
| --- | --- | --- | --- | --- | --- | --- | --- | --- | --- |
| Weight | N | True log HR | Bias of  log HR | Emp SE | Uncorrected  variance | Corrected variance |  | Uncorrected  variance | Corrected variance |
| ATE | 300 | 0.2300 | -0.007 | 0.150 | 0.225 | 0.148 |  | 0.993 | 0.947 |
| ATE | 1000 | 0.2300 | 0.000 | 0.073 | 0.120 | 0.075 |  | 0.997 | 0.957 |
| ATE | 10000 | 0.2300 | 0.000 | 0.023 | 0.038 | 0.023 |  | 0.999 | 0.947 |
| ATT | 300 | 0.1622 | 0.001 | 0.155 | 0.225 | 0.152 |  | 0.991 | 0.941 |
| ATT | 1000 | 0.1622 | 0.000 | 0.085 | 0.124 | 0.084 |  | 0.996 | 0.942 |
| ATT | 10000 | 0.1622 | 0.000 | 0.028 | 0.039 | 0.026 |  | 0.998 | 0.941 |
| ATO | 300 | 0.1695 | -0.009 | 0.158 | 0.224 | 0.147 |  | 0.991 | 0.919 |
| ATO | 1000 | 0.1695 | -0.003 | 0.085 | 0.123 | 0.081 |  | 0.990 | 0.942 |
| ATO | 10000 | 0.1695 | -0.001 | 0.026 | 0.039 | 0.026 |  | 0.995 | 0.938 |

Abbreviations: Emp SE, empirical standard error; HR, hazard ratio; log, natural logarithm; N, sample size, SE, standard error

**Table S8** Simulation results for scenario 9

|  |  |  |  |  | Mean of SE | |  | Coverage probability | |
| --- | --- | --- | --- | --- | --- | --- | --- | --- | --- |
| Weight | N | True log HR | Bias of  log HR | Emp SE | Uncorrected  variance | Corrected variance |  | Uncorrected  variance | Corrected variance |
| ATE | 300 | 0.2303 | 0.046 | 0.275 | 0.275 | 0.214 |  | 0.920 | 0.862 |
| ATE | 1000 | 0.2303 | 0.013 | 0.148 | 0.163 | 0.132 |  | 0.960 | 0.913 |
| ATE | 10000 | 0.2303 | 0.001 | 0.047 | 0.054 | 0.045 |  | 0.981 | 0.943 |
| ATT | 300 | 0.1574 | -0.008 | 0.127 | 0.197 | 0.122 |  | 0.996 | 0.936 |
| ATT | 1000 | 0.1574 | 0.001 | 0.070 | 0.108 | 0.066 |  | 0.992 | 0.942 |
| ATT | 10000 | 0.1574 | 0.000 | 0.021 | 0.034 | 0.021 |  | 0.999 | 0.944 |
| ATO | 300 | 0.1692 | 0.002 | 0.138 | 0.201 | 0.130 |  | 0.990 | 0.936 |
| ATO | 1000 | 0.1692 | -0.005 | 0.074 | 0.111 | 0.072 |  | 0.994 | 0.949 |
| ATO | 10000 | 0.1692 | -0.001 | 0.023 | 0.035 | 0.023 |  | 0.994 | 0.944 |

Abbreviations: Emp SE, empirical standard error; HR, hazard ratio; log, natural logarithm; N, sample size, SE, standard error

**Table S9** Simulation results for scenario 10

|  |  |  |  |  | Mean of SE | |  | Coverage probability | |
| --- | --- | --- | --- | --- | --- | --- | --- | --- | --- |
| Weight | N | True log HR | Bias of  log HR | Emp SE | Uncorrected  variance | Corrected variance |  | Uncorrected  variance | Corrected variance |
| ATE | 300 | 0.2973 | -0.009 | 0.171 | 0.217 | 0.155 |  | 0.986 | 0.916 |
| ATE | 1000 | 0.2973 | 0.001 | 0.081 | 0.116 | 0.081 |  | 0.996 | 0.957 |
| ATE | 10000 | 0.2973 | 0.000 | 0.024 | 0.036 | 0.025 |  | 0.999 | 0.956 |
| ATT | 300 | 0.2094 | 0.000 | 0.179 | 0.217 | 0.172 |  | 0.977 | 0.941 |
| ATT | 1000 | 0.2094 | -0.001 | 0.093 | 0.120 | 0.094 |  | 0.992 | 0.960 |
| ATT | 10000 | 0.2094 | -0.001 | 0.029 | 0.038 | 0.029 |  | 0.991 | 0.958 |
| ATO | 300 | 0.2182 | 0.005 | 0.171 | 0.214 | 0.164 |  | 0.976 | 0.947 |
| ATO | 1000 | 0.2182 | 0.001 | 0.090 | 0.119 | 0.091 |  | 0.990 | 0.952 |
| ATO | 10000 | 0.2182 | 0.000 | 0.029 | 0.038 | 0.029 |  | 0.989 | 0.939 |

Abbreviations: Emp SE, empirical standard error; HR, hazard ratio; log, natural logarithm; N, sample size, SE, standard error

**Table S10** Simulation results for scenario 11

|  |  |  |  |  | Mean of SE | |  | Coverage probability | |
| --- | --- | --- | --- | --- | --- | --- | --- | --- | --- |
| Weight | N | True log HR | Bias of  log HR | Emp SE | Uncorrected  variance | Corrected variance |  | Uncorrected  variance | Corrected variance |
| ATE | 300 | 0.2978 | 0.047 | 0.281 | 0.262 | 0.214 |  | 0.899 | 0.854 |
| ATE | 1000 | 0.2978 | 0.011 | 0.159 | 0.162 | 0.137 |  | 0.938 | 0.894 |
| ATE | 10000 | 0.2978 | 0.001 | 0.048 | 0.054 | 0.047 |  | 0.972 | 0.945 |
| ATT | 300 | 0.2078 | -0.003 | 0.148 | 0.195 | 0.146 |  | 0.982 | 0.942 |
| ATT | 1000 | 0.2078 | 0.003 | 0.079 | 0.107 | 0.079 |  | 0.991 | 0.948 |
| ATT | 10000 | 0.2078 | -0.001 | 0.026 | 0.034 | 0.025 |  | 0.991 | 0.936 |
| ATO | 300 | 0.2219 | 0.011 | 0.160 | 0.200 | 0.151 |  | 0.979 | 0.929 |
| ATO | 1000 | 0.2219 | 0.004 | 0.083 | 0.110 | 0.084 |  | 0.990 | 0.961 |
| ATO | 10000 | 0.2219 | 0.000 | 0.027 | 0.035 | 0.027 |  | 0.990 | 0.953 |

Abbreviations: Emp SE, empirical standard error; HR, hazard ratio; log, natural logarithm; N, sample size, SE, standard error
